# The Relative Influence of Competition and Prey Defenses on the Phenotypic Structure of Insectivorous Bat Ensembles in Southern Africa

**DOI:** 10.1371/journal.pone.0003715

**Published:** 2008-11-13

**Authors:** M. Corrie Schoeman, David S. Jacobs

**Affiliations:** 1 Department of Zoology, University of Cape Town, Rondebosch, South Africa; 2 School of Biological and Conservation Sciences, University of KwaZulu-Natal, Durban, South Africa; Monterey Bay Aquarium Research Institute, United States of America

## Abstract

Deterministic filters such as competition and prey defences should have a strong influence on the community structure of animals such as insectivorous bats that have life histories characterized by low fecundity, low predation risk, long life expectancy, and stable populations. We investigated the relative influence of these two deterministic filters on the phenotypic structure of insectivorous bat ensembles in southern Africa. We used null models to simulate the random phenotypic patterns expected in the absence of competition or prey defences and analysed the deviations of the observed phenotypic pattern from these expected random patterns. The phenotypic structure at local scales exhibited non-random patterns consistent with both competition and prey defense hypotheses. There was evidence that competition influenced body size distribution across ensembles. Competition also influenced wing and echolocation patterns in ensembles and in functional foraging groups with high species richness or abundance. At the same time, prey defense filters influenced echolocation patterns in two species-poor ensembles. Non-random patterns remained evident even after we removed the influence of body size from wing morphology and echolocation parameters taking phylogeny into account. However, abiotic filters such as geographic distribution ranges of small and large-bodied species, extinction risk, and the physics of flight and sound probably also interacted with biotic filters at local and/or regional scales to influence the community structure of sympatric bats in southern Africa. Future studies should investigate alternative parameters that define bat community structure such as diet and abundance to better determine the influence of competition and prey defences on the structure of insectivorous bat ensembles in southern Africa.

## Introduction

One of the principal questions of community ecology is whether or not local communities are equilibrated and structured by deterministic processes such as competition. The alternative is that communities are not equilibrated and composed of species that co-occur purely by chance [1], [2]. Useful rules and generalizations of community structure often only emerge when a broad-scale or macroecological view is taken [3]. At a macroecological scale focus is less on the properties of single species and more on the emergent properties of community organization [4]. Within a macroecological framework, species composition on a local scale is viewed as a consequence of a multistage, multi-layered process that starts at the top with a regional species pool that extends over a much larger spatial scale than the local community. Species originate from the regional pool and pass through a series of environmental filters before establishing themselves as members of the local community [5]. These filters work on different spatial and temporal scales, and may overlap [6]. However, fierce debate on the relative roles of deterministic filters versus chance in community ecology has continued for almost three decades with no consensus in sight [5].

Community ecologists have no a priori way of knowing which environmental filters structure local communities [5]. Nonetheless, deterministic filters are more likely to structure communities in stable systems than they are in unstable systems [5], [7]. Specifically, competition should influence the community structure of animals such as bats with life histories characterized by low fecundity, low predation risk, long life expectancy, and stable populations [8]. Furthermore, influences of deterministic filters on communities are probably more species specific than the influences of abiotic filters. For example, species richness in most volant and non-volant mammal communities decreases with increasing latitude due mainly to lower temperatures [9]. Conversely, predation probably influences the community structure of small non-volant mammals more significantly than it would the community structure of small volant mammals (i.e. bats) [10], [11]. Moreover, small, non-volant mammals must meet their ecological requirements within a smaller spatial scale, leading to finer-grained and less specialized patterns of resource utilization than those of small volant mammals [12]. Thus, deterministic processes such as competition should be the most important filters structuring bat assemblages.

If competition influenced the phenotypic niche structure of bat assemblages, the following predictions can be made. Phenotypic distances between species should be greater than distances chosen at random from a particular distribution of distances [13]. Alternatively, phenotypic distances between species should be less variable—i.e. species should be more evenly spaced-than the variance of distances expected by chance [14]. Phenotypic traits of sympatric bat species that are most likely to be influenced by competition include body size, wing morphology, and echolocation [15]–[18].

If coevolution between bats and their food sources rather than interspecific competition influenced community structure then the phenotypic distances between coexisting bat species should be smaller than the distances between species expected by chance [19], [20]. The interaction between insectivorous bats and tympanate insects such as moths, is one of the most cited examples of a coevolutionary arms race [21]. Tympanate insects have auditory systems adapted to hear the echolocation calls of bats that prey on them, and the bats in response may have adapted their echolocation calls and/or foraging behaviour to overcome these defences. Consequently, peak echolocation frequency is often a better predictor of diet than size or wing parameters of insectivorous bats [22]–[24]. Thus, echolocation rather than body size or wing morphology should be significantly similar among coexisting bats if coevolution between bats and insects mediated by prey defences influenced bat community structure.

The few extensive broad-scale analyses that have investigated the influence of deterministic processes such as competition or coevolution on the community structure of multiple bat assemblages using, for example, null models have been restricted to New World faunas [12], [25]. These studies suggest that deterministic processes have only a limited influence on bat community structure [20], [26], [27], or are not consistent over biogeographic areas and feeding guilds [28], [29]. However, most of these studies considered bat assemblages in large areas of the New World, i.e. regional or gamma diversity, and may therefore not reflect co-occurring groups of species that interact at a particular habitat and time (local and alpha diversity) [30]. Furthermore, differences in the biogeographic histories of the Old and New Worlds mean these regions differ not only in the systematics of local bat faunas, but also in the degree to which various local and regional filters influence community structure [25].

In this study, we tested the contrasting predictions from the competition and prey defence hypotheses on the phenotypic structure of insectivorous bats at local and regional scales in southern Africa. We quantified phenotypic differences of body size, wing morphology and echolocation between coexisting species with two indices, minimum segment length ratio and variance of segment length ratios, and compared them with corresponding patterns expected by chance that were derived by random sampling from known and simulated regional source pools. We predicted that the minimum segment length ratios of phenotypic differences between coexisting bats should be significantly large, and the variance of segment length ratios should be significantly small, if competition influenced community structure. Conversely, if prey defence filters influenced community structure, we predicted that the minimum segment length ratios of echolocation rather than those of body size or wing morphology should be significantly small between coexisting species.

We use the term “ensemble” to describe a set of co-occurring species within an assemblage (i.e. bats) that belong to a common functional group [31], i.e. insectivorous bats. Grouping of very different entities such as assemblages, guilds, or ensembles under the umbrella term “community” may inhibit progress in understanding the dynamics of these complex ecological systems [12]. At the ensemble level of organization, biotic filters are expected to have a stronger effect on the morphological patterns of the species sets [30]. We therefore also classified the bat species of an ensemble into three functional foraging groups: open-air, clutter-edge, and clutter feeders [32]. The adaptive complex (sensu [33]) of size, wing morphology and echolocation clearly defines the niche and foraging behaviour of sympatric insectivorous bats into these three functional groups [32], [34]. Thus, member species of each functional group may be more likely to competitively interact with each other than with member species of other functional groups [17].

## Results

Size, wing, and echolocation parameters of 42 insectivorous bat species were measured (Table S1). Although four other species, *Mimetillus moloneyi*, *Nycteris hispida*, *Scotoecus albigula*, and *Scotophilus viridis* were captured during surveys at 16 other local sites in southern Africa, it was not possible to record their echolocation calls and were therefore not included in analyses. In any case, these species were very rare and were not recorded in the ensembles. The CFK regional species pool inventory totaled 13 insectivorous bat species representing 11 genera and five families, the Nama-Karoo regional species pool totaled 18 species representing 12 genera and six families, and the savanna regional species pool inventory totaled 37 insectivorous bat species representing 20 genera and seven families (Table S1). At a local scale, the savanna ensemble, SU, exhibited the highest species richness. Among CFK ensembles, species richness was highest at the AL ensemble, while species richness of the remaining three ensembles was markedly similar (Table S1). The accuracy of the observed species richness of ensembles and regional species pools was verified statistically using sample-based rarefaction and species richness estimators (Schoeman & Jacobs unpublished data).

### Principal component analyses

**Figure 1:**
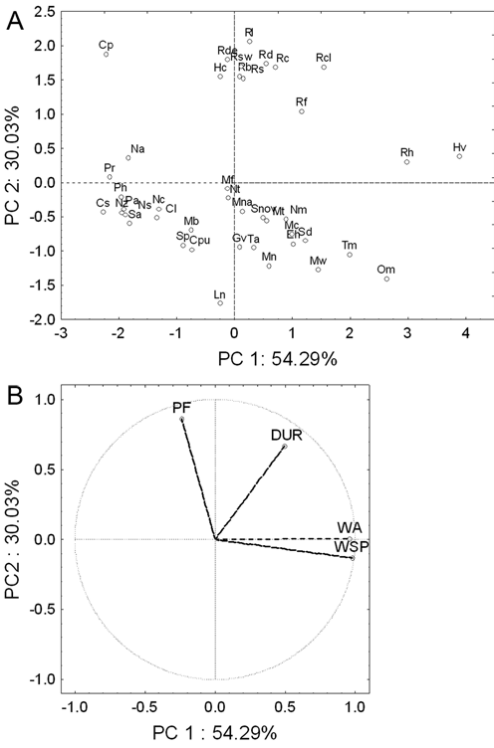
Principal component analysis of wing (WA and WSP) and echolocation (PF and DUR) parameters of 42 southern African insectivorous bat species. A. Plot of component scores of species on the first two principal components (PC1 and PC2). B. Plot of component weights for echolocation and wing parameters on the first two principal components. Dotted line shows distance to midpoint (0, 0) of graph.

The first two unrotated principal components (PCs) accounted for 84.3% of the total variance of the echolocation and wing morphology among the 42 species, and grouped species along family and functional foraging group divisions (Fig. 1A). The only exceptions were the open-air bats *Chaerephon pumilus* and *Sauromys petrophilus* that grouped with the clutter-edge bats and not with the open-air bat species. Plotting the factor loadings resulted in the clear separation of PF and DUR from the two wing parameters (Fig. 1B). Varimax rotation did not alter or clarify these patterns appreciably.

These patterns were interpreted as follows. Firstly, PC1 was a measure of differences in wing morphology and bats that loaded high on PC1 (e.g. *Hipposideros vitatus* and *Rhinolophus hildebrandti*) had wings with much larger WA and WSP than bats that loaded low on PC1 (e.g. *Cloetis percivali* and *Cistugo seabrai*; Table S1). Secondly, PC2 was a measure of differences in PF, and bats that loaded high on PC2 had echolocation calls of much higher PF (e.g. *Cloeotis percivali*: 208 kHz) than bats that loaded low on PC2 (Table S1).

First and second principal components of the PCAs for the three functional groups accounted for 46.4 to 75.5% and 22.5 to 27.7%, respectively, of the total variance of the echolocation and wing morphology among species (Table 1). Separation of bat species was less clear, and different parameters contributed to the first and second principal components. However, wing morphology was always linked to the first principal component and echolocation to the second principal component (Table 2).

**Table 1:** Eigenvalues and percent variation of the first two principal components (PCs) derived from principal component analyses of wing and echolocation parameters of bat species classified to three functional foraging groups.

Functional group	Spp richness	PC1		PC2	
		Eigenvalue	% Variation	Eigenvalue	% Variation
Clutter-edge	20	2.3	46.4	1.4	27.7
Clutter	15	2.6	65.8	1.1	26.7
Open-air	7	3.8	75.5	1.1	22.5

**Table 2:** of the first two principal components (PCs) derived from principal component analyses of wing (WSP = wingspan, and WA = wing area) and echolocation (PF = peak echolocation frequency, BW = bandwidth for low duty-cycle echolocation bats, and DUR = duration) parameters of bat species classified to functional foraging groups (boldface print indicates phenotypic characters contributing most to principal components).

	Functional foraging groups					
	Clutter-edge		Clutter		Open-air	
Parameter	PC1	PC2	PC1	PC2	PC1	PC2
WSP	**0.954**	0.086	**0.965**	−0.144	**0.986**	−0.154
WA	**0.958**	0.084	**0.965**	−0.205	**0.966**	−0.237
PF	−0.662	−0.14	**−0.866**	−0.231	−0.771	**−0.604**
BW	−0.202	**0.803**			−0.726	**0.676**
DUR	0.11	**−0.841**	0.139	**0.976**	**0.864**	0.471

### Non-random patterns predicted by competition

With the exception of GH, the variance of mass segment-length ratios between species was significantly smaller than expected by chance for all ensembles, irrespective of regional source pool used (Table S2). These non-random patterns indicate that mass was more evenly spaced among the species of ensembles than otherwise expected by chance. We also compared the mass segment-length ratios of the CFK and Nama-Karoo regional source pools to those assembled at random from the largest southern African regional source pool. Variance of segment-length ratios was significantly smaller than expected by chance in both biome regional pools (observed = 0.0006 and 0.002 versus expected = 0.009 and 0.006, respectively, p<0.001). Non-random patterns of mass were much less ubiquitous among species of the clutter-edge and clutter functional groups (Tables S3 and S4, respectively). Similarly, the variance of segment-length ratios among the four bat species caught at every ensemble, *Miniopterus natalensis* (Miniopteridae), *Neoromicia capensis* (Vespertilionidae), *Tadarida aegyptiaca* (Molossidae), and *Rhinolophus clivosus* (Rhinolophidae), was also not significantly smaller than expected by chance (observed = 0.004 versus expected = 0.007, 0.02, and 0.04 based on random sampling from the CFK, Nama-Karoo and southern African regional source pool, respectively, all p>0.05).

**Figure 2:**
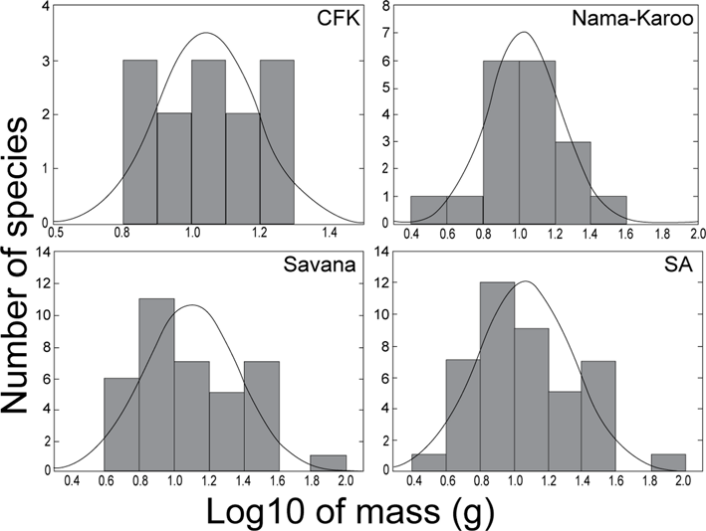
Hierarchical evaluation of the distribution of body sizes of insectivorous bat species at the scale of the Cape Floristic Kingdom (CFK), the Nama-Karoo, the savanna, and southern Africa (SA) source pools. The expected normal distribution curve is also shown.

**Figure 3:**
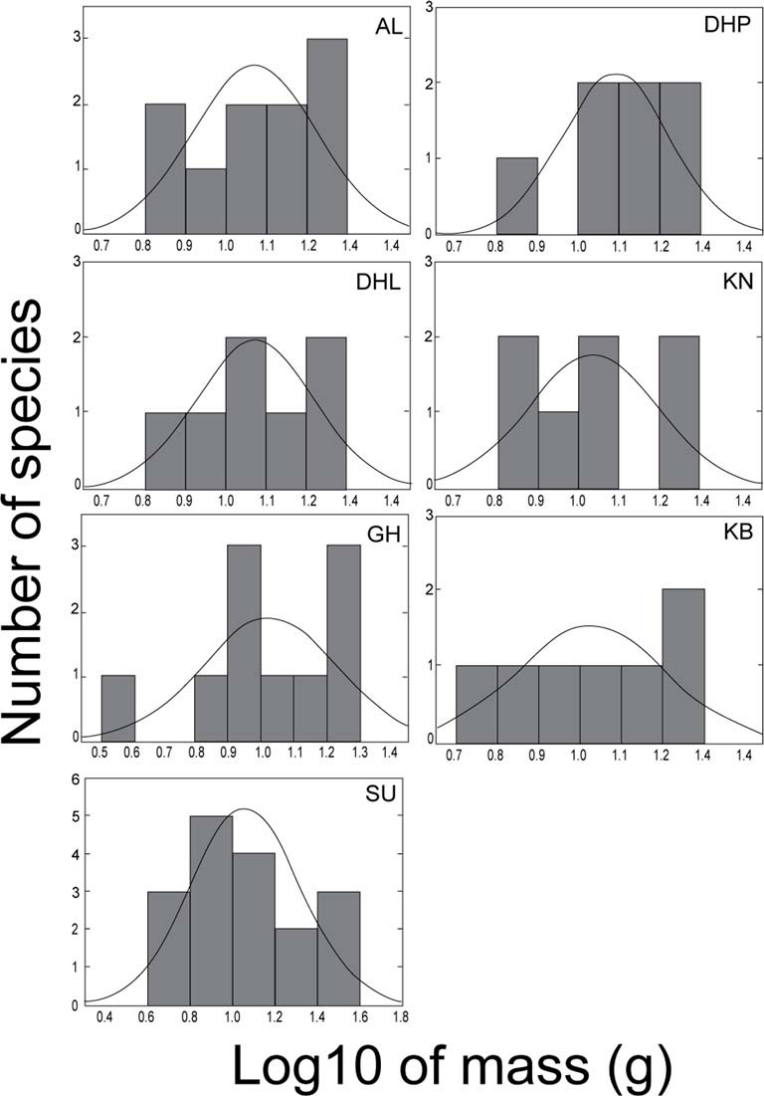
Distributions of body sizes of insectivorous bat species at the ensemble scale. The expected normal distribution curve is also shown..

Distribution of masses of bat species revealed different patterns at local and regional scales. Fig. 2 shows the distribution of masses of bat species classified to the regional source pools. At the scale of the largest regional source pool (all species caught in southern Africa), the distribution of masses on a logarithmic scale was unimodal and right-skewed (g_1_ = 0.64), but did not depart significantly from a log-normal distribution (Kolmogorov-Smirnov one sample test; d = 0.09, p = n.s.; Fig. 2). However, the distribution of masses appear scale dependant, becoming progressively more even, and less right-skewed, at savanna, Nama-Karoo, and CFK regional scales (g_1_ = 0.65,−0.1 and 0.15, respectively; Fig. 2), than distributions at the local scale (Fig. 3). The distribution of masses in the GH ensemble was clearly more random than in the other ensembles (Fig. 3), and therefore consistent with null model results. The random mass distribution of the coexisting bats at GH was linked to the presence of the small and very rare vespertilionid, *Cistugo seabrai* [58]. When we excluded *C. seabrai* from the GH matrix, and reanalysed the mass data, variance of segment-length ratios between species was significantly smaller than expected by chance (observed = 0.0009 versus expected = 0.013, p<0.001, based on random sampling from the SA regional source pool).

Only a few ensembles and functional groups displayed non-random distributions of principal component scores in the direction predicted by the competition hypotheses. Minimum PC1 segment-length ratios of the GH ensemble, and the SU clutter-edge and open functional groups, were significantly larger, and the variance of the PC1 segment ratios of the AL ensemble was significantly smaller, than expected by chance. Also, minimum PC2 segment-length ratios of the AL ensemble was significantly larger, and the variance of PC2 segment-length ratios between bats of the DHP clutter-edge functional group was significantly smaller, than expected by chance, based on biogeographic and log-uniform regional pools (Tables S2, S3, and S4). However, values of segment-length ratio indices of the CFK and Nama-Karoo regional pools were not significantly different from those expected by chance (all p>0.05).

### Non-random patterns predicted by prey defences

As predicted, minimum segment length ratios of mass and PC1 (i.e. wing morphology) never exhibited non-random patterns in the direction predicted by the prey defences hypothesis. Conversely, the minimum segment-length ratios of PC2 were significantly smaller than predicted by chance in the DHL and KN ensembles based on biogeographic and log-uniform regional pools (Table S2). Minimum segment-length ratios of the CFK, Nama-Karoo, and savanna regional source pools were not significantly smaller than predicted by chance (all p>0.05).

Comparative analyses of phenotypic characters need to account specifically for the effect of body size [38] and phylogeny [59]. Therefore, we repeated the PCAs and null model analyses after removing the influence of body size from wing morphology echolocation parameters while controlling for phylogeny (after [60]). However, non-random patterns of principal components consistent with competition and prey defence hypotheses were very similar to the non-random patterns discussed above, and are therefore not reported here.

## Discussion

Our null model analyses of the phenotypic structure of insectivorous bat ensembles and functional foraging groups found non-random patterns consistent with competition and prey defense hypotheses. However, the ensembles, functional foraging groups in ensembles, and functional foraging groups in general, differed in the manner and degree to which they were structured, and non-random patterns were ubiquitous only for body size.

### Non-random patterns predicted by competition hypotheses

Body size (mass) was evenly spaced at a local scale (i.e. every ensemble except GH), and at intermediate regional scales (the Cape Floristic Kingdom and Nama-Karoo biome regional source pools). Our findings are thus consistent with evidence from detailed studies of coexisting vertebrate species that show similar non-random patterns of body size (e.g. [61]–[65]). However, body size distribution of southern African insectivorous bats was scale-dependant, becoming progressively more right-skewed and less evenly spaced from local to regional levels. The right-skewed body size distribution at the greatest regional scale is consistent with body size distributions of non-volant and volant New World mammals at regional and continental scales [25], [66], [67]. Our results are also consistent with the observation that body sizes of non-volant mammals tend to be evenly distributed at a local scale [66], [67]. They differ, however, with body size patterns of Mexican bat assemblages where the frequency distribution of body sizes was right-skewed at local, intermediate and regional scale [67]. The inclusion of fruit-eating bats in the data matrix of the latter study may explain the right-skewed size distribution at local scale. Average body size of fruit-eating bats is significantly larger than average body size of insectivorous bats [68]. In contrast to New World volant and non-volant mammals [25], [66], [67], however, the range of southern African insectivorous bat body sizes was narrower at a local scale than at a regional scale. Small and large-bodied bat species caught in the sub-tropical savannas were absent from the temperate fynbos and forest ensembles. This suggests that abiotic filters rather than competition filters may have structured the distribution of mass in ensembles.

Principal component scores that were linked to wing morphology and echolocation of bat species displayed non-random patterns predicted by competition theory in species-rich ensembles and functional foraging groups. The savanna ensemble, SU, was significantly more species rich than the other ensembles, and the fynbos ensemble, AL, and the Nama-Karoo ensemble, GH, were significantly more species rich than the other CFK and Nama-Karoo ensembles. Competition theory predicts that interspecific competition is more likely among a large number of sympatric species that exceed the limit of similarity, than among a small number of similar species [13]. Furthermore, stochastic environmental processes may be less likely to override competitive interactions if the community is relatively species rich [69]. Similarly, more than 250 000 Miniopterus natalensis bats roost for c.a. eight months of the year in the fynbos ensemble DHP, consuming approximately 100 tons of insects [70]. Intraspecific variation in wingspan and echolocation flexibility enables the species to utilize open and cluttered habitats at DHP [71]. Consequently, resource utilization of the other coexisting bats, especially clutter-edge species, is probably severely affected. Thus, under conditions of high species richness or abundance, competition for resources might be strong enough to influence the flight and echolocation structure of coexisting bats.

Brown & Nicoletto [66] explained the differences in body size distribution between local and regional scales as an effect of local competitive exclusion, higher specialization of modal species, and higher extinction for large species with small range sizes. They propose that competitive exclusion is the most likely biotic process to explain why faunas at local scale harbour few modal-sized species and display an even distribution of body masses. If so, competitive interactions should be limited to those species utilizing similar food resources [3], [66]. However, we found no support for the prediction that non-random patterns should be more apparent within functional foraging groups. Instead, the limited range of body sizes at a local scale suggests that filters other than competitive interactions may prevent very small and very large-bodied species from establishing themselves in ensembles.

The fact that large- and small-bodied bat species were poorly represented in the regional source pools at intermediate scales suggests that there may be a low replacement rate of these species between the local and regional scale [66]. Consequently, certain large-scale abiotic processes may prevent the accumulation of small-bodied and/or large-bodied bat species in regional source pools [3], [66]. One hypothesized process is the selective extinction of species with large (or small) body sizes and small geographic ranges. Although our data do not directly test this hypothesis, there is evidence that lends some support. For example, the extinction risk of bat species is significantly correlated with small geographic ranges [72] and both large- and small-bodied species can have small geographic ranges. For example, large-bodied African bat species such as *Nycteris grandis* (39 g) and *Hipposideros vitatus* (68 g) have smaller geographic ranges than their smaller-bodied congenerics such as N. thebaica (13 g) and *H. caffer* (9 g), respectively [54], [70].

The physics of flight and sound combined with the small size of volant prey severely limits the viable body size range displayed by echolocating bats [41], [68]. The mechanics of prey capture in flight, coupled with the small effective range of echolocation, selects for a small body size capable of the maneuverability and agility necessary to hunt small, volant prey at short range [68]. Furthermore, the coupling of flight and echolocation mechanisms puts a lower limit on echolocation frequencies, and therefore an upper limit to body size, necessary to detect and catch small flying prey [41], [68]. Thus, the non-random phenotypic patterns of insectivorous bats at a local scale, and at an intermediate regional scale, may be an artifact of the constraints of flight and echolocation.

### Non-random patterns predicted by the prey defence hypothesis

In this study, sympatric bat species of two ensembles were more similar in echolocation parameters than expected by the null model. As we predicted, mass and wing morphology never exhibited non-random patterns consistent with the prey defence hypothesis. By comparison, temperate North American hummingbirds were more similar in mass, bill length, and wing length than predicted by null models (Bowers & Brown 1985). These morphological patterns were attributed to mutualist coevolutionary processes with flowers. Conversely, neither nectarivore nor foliage-gleaning bat ensembles from Caatinga or Cerrado in Brazil exhibited non-random patterns predicted by coevolutionary hypotheses [20]. If bats and their insect prey coevolved, there would have been stronger and more direct interaction, over evolutionary time, between insect hearing and bat echolocation than between insect hearing and the body size of bats. This is supported by studies that found that echolocation is a better predictor of diet than size or wing morphology of insectivorous bats [22]–[24].

There may be forces other than prey defences that promote animals to be more similar than expected by chance, however. If certain resource states are very abundant, for example, similar phenotypic patterns among species may be favoured because there is no competition for those resources [73]. Alternatively, 20 to 60 kHz is the peak frequency range used by most echolocating bats [21], because the frequency dependent effects of atmospheric attenuation and target strength mean that detection distance and target resolution is optimized within this frequency range [74]. Thus, non-random patterns consistent with predictions of the prey defence hypothesis may instead reflect the narrow but optimal range of echolocation frequencies that are used by sympatric insectivorous bats exploiting an abundant resource.

In summary, we found support for the predictions of both the competition and prey defense hypotheses. Nonetheless, the nature of this support is such that other factors cannot be excluded as being responsible for the non-random phenotypic structure of insectivorous bat ensembles in southern Africa. There was evidence that interspecific competition influenced body size at local and intermediate regional scales, and wing morphology and echolocation characters of ensembles and functional foraging groups with high species richness and abundance. There was also evidence that prey defences influenced the echolocation structure of two relatively species-poor ensembles. Evidence for these hypotheses was however lacking at other scales and in other functional foraging groups. Furthermore, abiotic filters such as geographic distribution ranges of small and large-bodied bat species, extinction risk, and the physics of flight and sound probably also interacted at local, regional and continental scales to influence the phenotypic structure of coexisting insectivorous bats at a local scale. This suggest that the life history characteristics of bats such as high vagility, combined with high levels of environmental heterogeneity and variability of the southern African landscape, prevent density-dependent interactions such as competition and prey defences from influencing the phenotypic structure in an ubiquitous fashion. Nonetheless, morphology is only one parameter defining community structure that can be influenced by competition and prey defense filters. These filters can give rise to non-random patterns of abundance and diet, for example, and these parameters should therefore also be examined - separately, and in relation to morphology (e.g. [29]) - to better determine the influence of competition and prey defences on the structure of insectivorous bat ensembles in southern Africa.

## Materials and Methods

### Study sites

**Figure 4:**
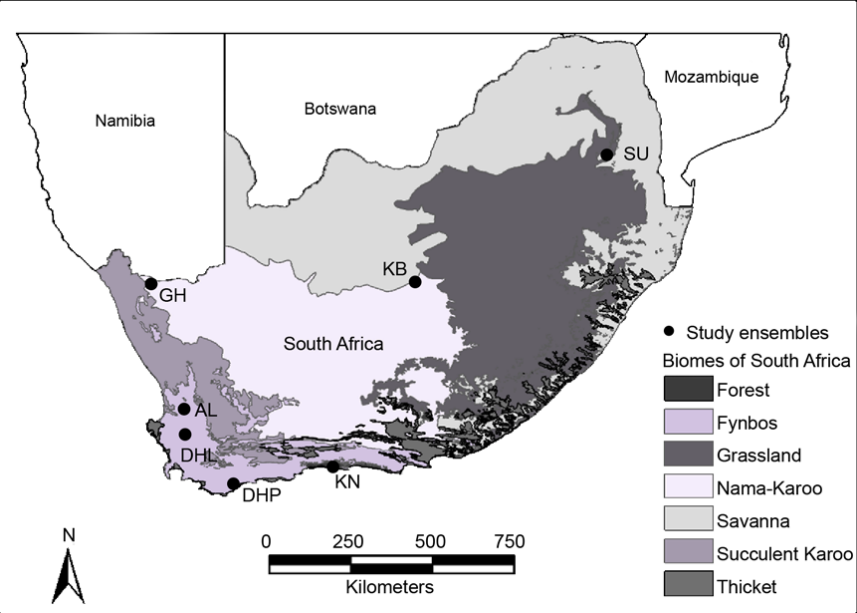
Distribution of biomes and political boundaries in southern Africa (biomes after [38]). Locations of the seven insectivorous bat ensembles are indicated with black markers.

Ensembles covered a delimited area where local bats had the potential to interact and were situated in or adjacent to nature reserves or conservancies to minimize the influence of urban development, agriculture, and alien fauna and flora on the local bat fauna. We sampled each of seven insectivorous bat ensembles as follows. Active and passive sampling methods took place at various trapping sites within a 10 km radius of the GPS coordinates taken at each of the ensembles (Fig. 4), with the exception of the forest ensemble near Knysna. Knysna sites were sampled in or near pockets of remaining forest from Rondevlei Nature Reserve in the west to Keurboomstrand in the east, a distance of c.a. 80 km. All ensembles, with the exception of the Nama-Karoo ensemble GH, were sampled during wet and dry seasons. The study ensembles and the methods used to survey their bat faunas are described in more detail in [35].

We sampled the bat faunas of three fynbos ensembles in the Cape Floristic Kingdom (CFK), Algeria Forestry Station (AL), Die Hel (DHL), and De Hoop Nature Reserve (DHP; Fig. 4). Each ensemble differed in their dominant type of vegetation, elevation, and mean annual rainfall. Fynbos vegetation dominates the CFK, and is characterized structurally by restioids, a high cover of ericoid shrubs, and an over-storey of proteoid shrubs [36]. Renosterveld, a low shrub layer dominated by *Elytropappus rhinocerotis*, a ground layer of grasses, and seasonally active geophytes, covers some 20 000 km^2^ of the CFK [36]. Fieldwork took place during winter and summer months in 2001–2004.

We sampled the bat fauna of one forest ensemble in the CFK, Knysna Forest (KN; Fig. 4). KN consists of relatively small pockets of indigenous forest, covering an area of 558 km^2^ along the southern coast from Mossel Bay to the Krom River and inland to the Outeniqua and Titsikamma Mountains. The forest has a closed canopy at an average height of 20 m. Tree composition, which includes Yellowwood (*Podocarpus folius*), hard pear (*Olinia ventosa*), and Stinkwood (*Ocotea bullata*), varies depending on the height above sea level, rainfall, type of soil, and slope [37]. Bats were sampled during winter and summer in 2003 and 2004, respectively.

We also sampled ensembles that occurred outside of the CFK, one in the savanna biome and two in the Nama-Karro biome. The savanna biome dominates the African continent [38], and covers 54% of southern Africa [39]. Vegetation can be varied but consists mainly of open woodland with mopane and Acacia trees, good grass cover, and various shrub species [39]. Sudwala Cave (SU, Fig. 4) is located 80 km from the Kruger National Park. Fieldwork at SU took place during summer and winter in 2002 and 2003.

The Nama-Karoo biome covers the central plateau of the western half of South Africa. It is the second-largest biome in southern Africa [38]. Vegetation in the Nama-Karoo biome is a combination of arid grassland and dwarf scrubland [40]. The Goodhouse (GH; Fig. 4) ensemble includes bats sampled at Gougap Nature Reserve (29°31′S, 18°00′E) and Goodhouse (28°56′S, 18°07′E) during the summer in 2002. The Koegelbeen cave (KB) is found within a sinkhole 25 km from Griekwastad near Kimberly. Fieldwork at KB took place during the summer in 1998 and during the winter in 2004.

We also surveyed bats at 16 other local sites in southern Africa [35]. This was necessary to generate regional source pools of the CFK, Nama-Karoo and savanna biome that included bat species not captured in the ensembles. Most of these sites were located in the species rich savanna biome.

### Body size, wing morphology and echolocation

Body mass (to nearest 0.5 g) of each captured bat was measured with a Pesola scale. Forearm length is a good measure of body size, and frequently used to compare interspecific differences of body size between bats (e.g [22], [41]). However, provided it is measured after the digestive tract of the bat has been voided, mass is a better measure of body size, because it is not dependent on wing shape or taxonomic affiliation of the species and therefore well suited to compare body size of different taxa [3]. Measurements from juveniles and gravid females were excluded to avoid biasing means of species.

Wing area and wingspan were used as measures of absolute wing size [34]. The extended right wing of each captured bat (after [42]) was photographed with an Olympus C730 digital camera (Olympus America Inc., New York, USA) ensuring that the camera was positioned at 90° above the wing. Each wing was extended at a similar angle flat on graph paper, and the right hind limb and tail membrane could be opened and secured in position to the graph paper with masking tape. These wing images were calibrated with the dimensions of the graph paper to measure wingspan (WSP, to nearest 0.1 mm) and wing area (WA, including body area without the head, and the area of the uropatagium (after [34]) to the nearest 0.1 mm2, using SigmaScan Pro 5 software (version 5.0.0, SPSS Inc., Aspire Software International, Leesburg, USA).

Echolocation signals of low duty-cycle echolocating bats were recorded from hand-released bats. Bats were followed for as long as possible after release to ensure that search phase calls were recorded [43]. Bats were released just before dusk the day after they were captured. This ensured that there was sufficient light for us to follow them and that there were no other species about. Bats were released in open spaces, at least 15 m from the nearest obstacles to minimize variability in signal parameters due to proximity to obstacles [44]. Echolocation signals of high duty-cycle echolocating bats (i.e. Rhinolophidae and Hipposideridae) were recorded with the bat held in the hand, eliminating any possible variation in frequency as a result of Doppler shift compensation by the bat when in flight [16]. Each bat was released in the habitat in which we caught it. We used duration (DUR), bandwidth (BW), and peak echolocation frequency (PF) for low duty-cycle echolocating bats. We only used DUR and PF for high duty-cycle echolocating bats.

Echolocation calls of bats were recorded on a Compaq Presario 1400 personal computer using a DAQ 6062E high speed sound card (National Instruments, Austin, Texas) connected to the high frequency output of a Pettersson D980 bat detector (Pettersson Electronik AB, Uppsala, Sweden) via an anti-aliasing filter (F2000, Pettersson Electronik AB, Uppsala, Sweden). The resultant wave file was analysed using BatSound Pro software (version 3.20; Pettersson Elektronik AB, Uppsala, Sweden). The sampling frequency was set at 500 000 Hz (16 bits, mono), with a threshold of 16. One signal pulse for each bat was randomly selected to avoid pseudo-replication. Choice of signal pulse was subject to the following three criteria. First, signals with a high signal-to-noise ratio, i.e. the signal from the bat was at least three times stronger than the background noise as displayed on the time-amplitude window. Second, only signals that were not saturated were analyzed [45]. Finally, for low duty-cycle echolocating bats, only search phase signals that were recorded at least three seconds after releasing the bat were considered. The dominant harmonic from each call was taken from the Fast Fourier Transform (FFT) power spectrum [46]; size 512). A Hanning window was used to eliminate effects of background noise. PF was measured from the peak of the power spectrum [46]. BW was measured in signals of low duty-cycle echolocating bats at ±18 dB from the PF on the FFT power spectrum [47]. DUR was measured from the time-amplitude display [48].

### Species identification and means

Genetic analyses of wing tissue samples taken from captured bats with a 3 mm biopsy punch confirmed species identification [35], [49], [50]. The punctures clearly marked the bats and ensured that recaptures were not included in subsequent analyses.

Where possible, 10 individuals (five males and five females) were randomly selected to represent a species' mean. If fewer than five individuals of either sex were caught, or fewer than 10 individuals in total, all the individuals available were selected. Averaging parameters for males and females may represent a phenotype that does not occur in nature [51]. On the other hand, if each sex is treated as two morphospecies when using null models, the results will be difficult to interpret because the overlap within a species may not be statistically or biologically equivalent to overlap between species [51]. Furthermore, small sample sizes for some species precluded us from treating each sex separately. However, preliminary ANOVA analyses detected only limited sexual dimorphism in some species. Small sample sizes also precluded us from taking geographic variation of parameters into account. Using source pool averages for geographically highly variable species may reduce the size of the source pool if the average parameter in the source pool characterizes only a small fraction of all the populations representing the pool [51]. However, preliminary ANOVA analyses detected limited geographic variation in widespread species such as *Neoromicia capensis* and *Nycteris thebaica*. Thus, the average mass, WSP, WA, PF, BW (for low duty-cycle echolocating bats), and DUR were used for each species.

### Testing the predictions of competition and prey defense hypotheses

#### Phenotypic structure

We tested the predictions of competition and prey defense hypotheses on body size, wing morphology and echolocation characteristics (see above). We Log10 transformed these phenotypic characters to enhance normality and equalize variances. We created multivariate plots of wing morphology and echolocation using principal component analysis (PCA, Statistica version 7, Statsoft) such that distances between any two species on these plots were representative of the wing and echolocation differences between them [20]. PCA maintained morphological distances among species, yet eliminated redundancy of the highly correlated wing and echolocation characteristics that are part of the same adaptive complex [33]. For example, a wing shape that allowed fast flight would be useless if coupled with echolocation calls that only permit short detection distances. Such a bat would not be able to detect prey soon enough to capture them. Conversely, wing morphology adapted for slow maneuverable flight in clutter needs to be coupled with echolocation signals suitable for distinguishing between prey and clutter echoes. Consequently, the number of dimensions necessary to illustrate wing morphology and echolocation relationships was less than the original number of characters.

#### Segment-length ratio indices

Segment-length ratio indices are more appropriate than indices of absolute distance when testing predictions of competition hypotheses on size or morphology patterns [52]. Minimum segment-length ratio (MSL) was the segment-length ratio between the two species nearest in morphospace, i.e. the smallest segment-length ratio among the set of segment-length ratios. This index allowed us to test Hutchinson's [13] prediction that there should be a minimum spacing between species if competition structured the phenotypic niche of ensembles or functional groups. It also allowed us to test the contrasting prediction of the prey defense hypothesis that the two species should be closer in morphospace than otherwise expected [52]. If the MSL between species was significantly larger than 95% of simulated MSLs, we concluded that competition influenced the phenotypic structure of an ensemble or functional group. Conversely, if the MSL between species was significantly smaller than 95% of random values, we concluded that prey defences structured the phenotypic niche.

The variance of segment-length ratios among adjacent species in ensembles or functional groups, tested the prediction that species should be regularly spaced if competition influenced the phenotypic niche [52], [53]. Thus, if the observed variance was significantly smaller than 95% of simulated values, we concluded that competition structured the phenotypic niche of ensembles or functional groups.

#### Regional source pools

Values of segment-length indices calculated for observed ensembles were compared with values calculated for simulated ensembles that were assembled at random from regional source pools. Because no a priori regional pool size is preferred [28], [51], we used two different regional source pools for each ensemble. The first regional source pool included bat species whose distribution overlapped in the biome in which the ensemble occurs—based on distribution records [54], [55], and personal capture records. Hence, the CFK ensembles were compared with simulated ensembles drawn from the CFK regional pool (13 species), the Nama-Karoo ensembles with simulated ensembles drawn from the Nama-Karoo regional pool (17 species), and the savanna ensemble with simulated ensembles drawn from the savanna regional pool (38 species). The second regional pool included all the species caught in southern Africa.

#### Simulations and statistics

Using the Size Ratio module of Ecosim null model software (version 7.7, [52]), we statistically compared segment-length indices of observed ensembles and functional groups with those of simulated ensembles and functional groups assembled at random from the regional source pools. Simulated ensembles or functional groups were constructed by drawing the same number of species present in the observed ensemble or functional group, at random from the regional source pool. Species in regional source pools were drawn with equal probability. Once drawn, species could not be drawn again for that particular simulated ensemble or functional group. Minimum segment length ratios and variances were calculated for every simulated ensemble or functional group.

For each ensemble and regional species pool, we calculated the number of simulation ensembles or functional groups that could be assembled from the algorithm:
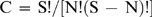
where, C is the number of ensembles or groups, N is the number of species in the ensemble or functional group, and S is the number of species in the regional source pool [20]. C was often very large so when C>1000, we selected a random 1000 simulated ensembles or functional foraging groups to calculate probability statistics. When C<1000, we calculated statistics based on the actual number of simulations possible.

To test if results from the above null models were specific to the regional source pools that were used, we compared the segment-length values of ensembles and regional source pools with those sampled randomly from a log-uniform null distribution, i.e. where there were approximately equal numbers of species in each of the segment-length ratio classes [52]. The endpoints of the log-uniform null distribution were fixed by the largest and the smallest bat species caught during the study. Ecosim generated a set of default values: the default minimum was 10% less than the observed minimum, and the default maximum was 10% more than the observed maximum [52]. For 1000 simulations, Ecosim randomly and uniformly selected a point greater than or equal to the smallest boundary and less than or equal to the largest boundary, for n species in an ensemble or regional source pool.

If more than 95% of the minimum segment-length or variance in segment-lengths of the simulated ensembles were larger or smaller than the observed ensemble, we concluded that patterns of the observed ensemble were non-random [56]. In addition, experiment-wise error of the significance tests (i.e. p values) was held constant at five percent for ensembles separately from functional groups at each site by application of Bonferroni sequential adjustments [57].

## Supporting Information

Table S1 Mean ±SD mass, wing (WSP = wingspan, and WA = wing area), and echolocation (PF = peak echolocation frequency, BW = bandwidth for low duty-cycle echolocation bats, and DUR = duration) parameters of 42 insectivorous bat species caught in southern Africa.
(0.20 MB DOC)

Table S2 Observed and expected segment-length ratio indices-minimum segment-length (MSL) and variance of segment length ratios-of body size (mass) and principal component (PC1 and PC2) parameters of bats caught in the fynbos (AL, DHL, DHP), forest, (KN), Nama-Karoo (GH and KB), and savanna (SU) ensembles.
(0.14 MB DOC)

Table S3 Observed and expected segment-length ratio indices-minimum segment-length (MSL) and variance of segment length ratios-of body size (mass) and principal component (PC1 and PC2) parameters of clutter (CLUT), and open-air (OPEN) bats caught in the fynbos (AL, DHP), forest, (KN), Nama-Karoo (GH, KB), and savanna (SU) ensembles.
(0.10 MB DOC)

Table S4 Observed and expected segment-length ratio indices-minimum segment-length (MSL) and variance of segment length ratios-of body size (mass) and principal component (PC1 and PC2) parameters of clutter-edge bats caught in the fynbos (AL, DHL, DHP), forest, (KN), Nama-Karoo (GH, KB), and savanna (SU) ensembles.
(0.11 MB DOC)
