# Supplementary material for: The Relative Influence of Competition and Prey Defenses on the Phenotypic Structure of Insectivorous Bat Ensembles in Southern Africa
Source: PLoS One. 2008 Nov 13;3(11):e3715. doi: 10.1371/journal.pone.0003715 (PMC2579324; doi:10.1371/journal.pone.0003715)
Supplement: Table S1 — Mean ±SD mass, wing (WSP = wingspan, and WA = wing area), and echolocation (PF = peak echolocation frequency, BW = bandwidth for low duty-cycle echolocation bats, and DUR = duration) parameters of 42 insectivorous bat species caught in southern Africa. (0.20 MB DOC) [file pone.0003715.s001.doc]

**Table S1** Mean ±SD mass, wing (WSP = wingspan, and WA = wing area), and echolocation (PF = peak echolocation frequency, BW = bandwidth for low duty-cycle echolocation bats, and DUR = duration) parameters of 42 insectivorous bat species caught in southern Africa.

|  |  |  | | Wing parameters | | | | Echolocation parameters | | | | | |  |  |  |
| --- | --- | --- | --- | --- | --- | --- | --- | --- | --- | --- | --- | --- | --- | --- | --- | --- |
|  |  | Mass (g) | | WSP (cm) | | WA (cm^2^) | | PF (kHz) | | BW (kHz) | | DUR (ms) | |  |  |  |
| Species | n | Mean | ±SD | Mean | ±SD | Mean | ±SD | Mean | ±SD | Mean | ±SD | Mean | ±SD | Ensemble | Biome | FG |
| *Chaerephon pumilus* | 10 | 11.2 | 1.3 | 26.6 | 1.2 | 96.2 | 9.3 | 29.9 | 1.5 | 15.7 | 2.3 | 4.1 | 0.1 | SU | N S | O |
| *Cistugo lesueuri* | 5 | 6.7 | 1.2 | 24 | 0.9 | 93.2 | 1.1 | 46.5 | 1.8 | 45.8 | 22.7 | 2.9 | 0.2 | AL | C N | CE |
| *Cistugo seabrai* | 2 | 3.8 | 0.4 | 21.2 | 1.1 | 65.2 | 7.3 | 45.8 | 0.7 | 24.4 | 10.8 | 3.1 | 0.7 | GH | N | CE |
| *Cloeotis percivali* | 6 | 5.3 | 0.6 | 20.9 | 1.3 | 83.7 | 5.2 | 207.8 | 3 |  |  | 4.6 | 1.2 | SU | S | C |
| *Eptesicus hottentotus* | 10 | 18.1 | 1.5 | 33.1 | 0.6 | 180.8 | 13.3 | 30.6 | 1.7 | 16.8 | 5.5 | 5.5 | 2.1 | AL, GH | C N S | CE |
| *Glauconycteris variegatus* | 1 | 13 |  | 30.4 |  | 148.4 |  | 41.1 |  | 21.1 |  | 2.3 |  |  | S | CE |
| *Hipposideros caffer* | 10 | 8.5 | 1.1 | 27.7 | 1.4 | 139.6 | 10.6 | 142.3 | 0.6 |  |  | 8.4 | 0.7 | GH, SU | N S | C |
| *Hipposideros vitatus* | 2 | 68.7 | 9.4 | 52.4 | 1.2 | 438.2 | 44.9 | 65 | 0.2 |  |  | 13.1 | 0.1 |  | S | C |
| *Hypsugo anchietai* | 1 | 6 |  | 21.7 |  | 86.9 |  | 55.9 |  | 25.2 |  | 2.1 |  | SU | S | CE |
| *Laephotus wintoni* | 5 | 10.3 | 1.6 | 27.4 | 1 | 134.4 | 14.6 | 22.4 | 0 | 16.3 | 0.7 | 2.1 | 0 | AL | C | CE |
| *Miniopterus fraterculus* | 10 | 8.9 | 0.5 | 29.4 | 1.2 | 136.5 | 8.9 | 62.1 | 1.5 | 17.2 | 6.3 | 3.7 | 0.7 | DHL, KN | C N S | CE |
| *Miniopterus natalensis* | 10 | 11.6 | 0.8 | 30.6 | 1.3 | 146 | 7.8 | 51.4 | 1.1 | 13.1 | 3.4 | 3.4 | 0.3 | AL, DHL, DHP, KN, SU, GH, KB | C N S | CE |
| *Mops condylurus* | 10 | 28.3 | 5.6 | 33.6 | 1.1 | 149.7 | 5.8 | 26.7 | 0.9 | 9.2 | 1.9 | 10 | 1.6 |  | S | O |
| *Mops niveiventer* | 6 | 28.7 | 2 | 31.7 | 1.3 | 129.7 | 10.9 | 20.3 | 0.2 | 10.6 | 0.8 | 8.1 | 0.6 |  | S | O |
| *Myotis bocagei* | 5 | 9.1 | 0.8 | 25.8 | 0.9 | 120.7 | 9.9 | 44.6 | 2.4 | 23.6 | 2.9 | 2.5 | 0.5 |  | S | CE |
| *Myotis tricolor* | 10 | 13.1 | 2.2 | 31.8 | 1.6 | 175.7 | 18.9 | 47.8 | 3.1 | 46 | 23.9 | 3.3 | 0.6 | AL, DHL, DHP, SU | C N S | CE |
| *Myotis welwitschii* | 1 | 18 |  | 36.6 |  | 234.9 |  | 34 |  | 16.4 |  | 2.4 |  |  | S | CE |
| *Neoromicia africanus* | 10 | 4.2 | 0.3 | 21.4 | 0.9 | 83.5 | 5.8 | 69.4 | 1.5 | 11.8 | 5.7 | 4.6 | 0.9 | SU | S | CE |
| *Neoromicia capensis* | 10 | 7.3 | 0.9 | 22.6 | 1 | 93 | 6.6 | 39.4 | 1.6 | 14.4 | 3 | 5.1 | 1.3 | AL, DHL, DHP, KN, SU, GH, KB | C N S | CE |

**Table S1** (Continued)

| *Neoromicia zuluensis* | 2 | 5.8 | 0.4 | 21.4 | 1.2 | 81.4 | 1.9 | 48.4 | 1.1 | 19.9 | 17.7 | 2.7 | 0.4 | SU | S | CE |
| --- | --- | --- | --- | --- | --- | --- | --- | --- | --- | --- | --- | --- | --- | --- | --- | --- |
| *Nycteris macrotis* | 2 | 17.8 | 0.4 | 35.1 | 1 | 237.3 | 9.5 | 76.7 | 13.2 | 20.3 | 0.4 | 1.2 | 0.5 |  | S | C |
| *Nycteris thebaica* | 10 | 12.6 | 1.5 | 29.3 | 1.6 | 167.3 | 9.8 | 77.5 | 10.3 | 30.2 | 11.9 | 1.7 | 0.2 | AL, DHP, SU, GH | C N S | C |
| *Nycticeinops schlieffeni* | 2 | 6.3 | 0.4 | 21.4 | 0.1 | 78.2 | 1.8 | 42.5 | 0.3 | 16.1 | 1.3 | 3.5 | 0.2 |  | S | CE |
| *Otomops martiensseni* | 2 | 32 | 2.8 | 40.4 | 0.1 | 209.2 | 12.6 | 11.8 | 0.3 | 7.8 | 0.3 | 27 | 1.4 |  | S | O |
| *Pipistrellus hesperidus* | 10 | 7.3 | 0.6 | 21.4 | 0.8 | 84.4 | 4.6 | 59.1 | 1.5 | 30.1 | 6.8 | 2.5 | 0.2 | KN, SU | C S | CE |
| *Pipistrellus rusticus* | 10 | 6.3 | 1.5 | 20.1 | 0.8 | 74.2 | 5.8 | 55.7 | 2.9 | 15.9 | 7.6 | 4.5 | 2.2 |  | S | CE |
| *Rhinolophus blasii* | 2 | 10 | 1.4 | 27 | 1 | 130.3 | 8.4 | 86.5 | 0.6 |  |  | 27.7 | 12.6 | SU | S | C |
| *Rhinolophus capensis* | 10 | 10.8 | 0.6 | 29.7 | 1.6 | 147.3 | 12.9 | 83.8 | 0.8 |  |  | 41.2 | 3.2 | DHL, DHP, KN | C N | C |
| *Rhinolophus clivosus* | 10 | 19 | 2.5 | 33.6 | 1.2 | 204.3 | 14.7 | 91.7 | 1 |  |  | 37.4 | 6.2 | AL, DHL, DHP, KN, SU, GH, KB | C N S | C |
| *Rhinolophus darlingi* | 10 | 8.8 | 1.6 | 28.5 | 1.3 | 146.1 | 11.8 | 87.1 | 2.1 |  |  | 39.5 | 10.6 | GH, KB, SU | N S | C |
| *Rhinolophus denti* | 10 | 5.9 | 0.2 | 27.1 | 0.8 | 123 | 7 | 111.2 | 1.8 |  |  | 23.4 | 4 | KB | N | C |
| *Rhinolophus fumigatus* | 2 | 12.3 | 0.4 | 30.8 | 2.1 | 169.6 | 37.5 | 53.7 | 0.2 |  |  | 40.3 | 0.9 |  | N S | C |
| *Rhinolophus hildebrandti* | 10 | 28.9 | 1.4 | 40.1 | 1.5 | 287.8 | 20.4 | 33.2 | 0.8 |  |  | 44.8 | 5.7 | SU | S | C |
| *Rhinolophus landeri* | 2 | 7.5 | 0.7 | 27 | 0.1 | 140.1 | 0.1 | 107.3 | 2 |  |  | 40 | 14.5 |  | S | C |
| *Rhinolophus simulator* | 10 | 8.2 | 1.4 | 26.9 | 1.1 | 132.1 | 8.4 | 80.1 | 1.2 |  |  | 31.3 | 7.5 | SU | S | C |
| *Rhinolophus swinnyi* | 10 | 7.7 | 0.5 | 27.9 | 0.6 | 135.3 | 5.2 | 106.6 | 0.4 |  |  | 22.3 | 3.3 |  | S | C |
| *Sauromys petrophilus* | 10 | 9.8 | 0.8 | 26 | 1.1 | 88.9 | 11.3 | 29.3 | 0.6 | 13.9 | 4.1 | 4.7 | 2.5 | AL, GH | C N S | O |
| *Scotoecus albofuscus* | 1 | 10.5 |  | 21.1 |  | 84 |  | 39.3 |  | 24 |  | 3.3 |  |  | S | CE |
| *Scotophilus dinganii* | 10 | 28.7 | 5,9 | 34.3 | 1.1 | 200.4 | 20.8 | 33.6 | 1.3 | 16.8 | 4.9 | 4.9 | 0.6 | SU | N S | CE |
| *Scotophilus sp. nov.* | 10 | 27.8 | 5.7 | 30.8 | 1.4 | 172.1 | 12.5 | 45.3 | 1.2 | 19.6 | 5.3 | 3.9 | 0.9 |  | S | CE |
| *Tadarida aegyptiaca* | 10 | 16 | 1.4 | 30.7 | 1.2 | 116 | 10.7 | 22.7 | 2.2 | 11.7 | 6.1 | 9.6 | 3.4 | AL, DHL, DHP, KN, SU, GH, KB | C N S | O |
| *Taphozous mauritianus* | 6 | 34 | 5 | 39.5 | 1.3 | 216.2 | 15.7 | 25.9 | 0.9 | 2.8 | 0.9 | 7.4 | 4.6 | SU | S | O |

Ensemble: AL = Algeria, DHL = Die Hel, DHP = De Hoop, KN = Knysna, GH = Goodhouse, KB = Koegelbeen, SU = Sudwala

Biome: C = Cape Floristic Kingdom, N = Nama-Karoo, S = savanna

Functional foraging group (FG): O = open-air, CE = clutter-edge, and C = clutter
