# Supplementary material for: The Relative Influence of Competition and Prey Defenses on the Phenotypic Structure of Insectivorous Bat Ensembles in Southern Africa
Source: PLoS One. 2008 Nov 13;3(11):e3715. doi: 10.1371/journal.pone.0003715 (PMC2579324; doi:10.1371/journal.pone.0003715)
Supplement: Table S2 — Observed and expected segment-length ratio indices-minimum segment-length (MSL) and variance of segment length ratios-of body size (mass) and principal component (PC1 and PC2) parameters of bats caught in the fynbos (AL, DHL, DHP), forest, (KN), Nama-Karoo (GH and KB), and savanna (SU) ensembles. (0.14 MB DOC) [file pone.0003715.s002.doc]

**Table S2** Observed and expected segment-length ratio indices - minimum segment-length (MSL) and variance of segment length ratios - of body size (mass) and principal component (PC1 and PC2) parameters of bats caught in the fynbos (AL, DHL, DHP), forest, (KN), Nama-Karoo (GH and KB), and savanna (SU) ensembles.

|  |  |  | AL | | DHL | | DHP | | KN | | GH | | KB | | SU | |
| --- | --- | --- | --- | --- | --- | --- | --- | --- | --- | --- | --- | --- | --- | --- | --- | --- |
| Parameter | Source Pool | Index | Obs | Exp | Obs | Exp | Obs | Exp | Obs | Exp | Obs | Exp | Obs | Exp | Obs | Exp |
| Mass | Biome | MSL | 0.02 | 0.02 | 0.03 | 0.02 | 0.02 | 0.03 | 0.03 | 0.03 | 0.02 | 0.02 | 0.04 | 0.03 | **0.009*** | 0.003 |
|  | Biome | Variance | 0.001 | 0.001 | **0.0005*** | 0.003 | 0.003 | 0.003 | 0.001 | 0.004 | 0.007 | 0.005 | **0.0008*** | 0.008 | **0.002*** | 0.006 |
| PC1 | Biome | MSL | 0.01 | 0.004 | 0.02 | 0.02 | 0.02 | 0.02 | 0.03 | 0.02 | **0.02*** | 0.002 | 0.001 | 0.01 | 0.003 | 0.002 |
|  | Biome | Variance | 0.002* | 0.007 | 0.01 | 0.01 | 0.01 | 0.01 | 0.02 | 0.01 | 0.05 | 0.008 | 0.01 | 0.008 | 0.007 | 0.009 |
| PC2 | Biome | MSL | 0.002* | 0.0004 | 0.0003 | 0.002 | 0.0004 | 0.003 | 0.0003 | 0.003 | 0.003 | 0.003 | 0.005 | 0.005 | 0.0009 | 0.001 |
|  | Biome | Variance | 0.02 | 0.009 | 0.008 | 0.01 | 0.01 | 0.01 | 0.009 | 0.01 | 0.007 | 0.006 | 0.01 | 0.01 | 0.002 | 0.001 |
| *C* |  |  |  | *78* |  | *1716* |  | *1716* |  | *1716* |  | *1.6E+7* |  | *1.6E+7* |  | *3E+7* |
| Mass | SA | MSL | **0.02*** | 0.007 | 0.03 | 0.02 | 0.02 | 0.02 | 0.03 | 0.02 | 0.02 | 0.008 | 0.04 | 0.02 | **0.009*** | 0.003 |
|  | SA | Variance | **0.001*** | 0.01 | **0.0005*** | 0.02 | **0.003*** | 0.02 | **0.001*** | 0.02 | 0.007 | 0.01 | **0.0008*** | 0.02 | **0.002*** | 0.006 |
| PC1 | SA | MSL | 0.01* | 0.003 | 0.02 | 0.01 | 0.02 | 0.01 | 0.03 | 0.02 | **0.02*** | 0.007 | 0.001 | 0.01 | 0.003 | 0.001 |
|  | SA | Variance | 0.002 | 0.004 | 0.01 | 0.02 | 0.01 | 0.02 | 0.02 | 0.02 | 0.05 | 0.02 | 0.01 | 0.02 | 0.007 | 0.009 |
| PC2 | SA | MSL | 0.002 | 0.003 | 0.0003 | 0.007 | 0.0004 | 0.008 | **0.0003^#^** | 0.008 | 0.003 | 0.003 | 0.005 | 0.008 | 0.0009 | 0.0009 |
|  | SA | Variance | 0.008 | 0.004 | 0.008 | 0.008 | 0.01 | 0.008 | 0.009 | 0.008 | 0.007 | 0.004 | 0.01 | 0.008 | 0.002 | 0.002 |
| *C* |  |  |  | *3.9E+8* |  | *3.2E+7* |  | *3.2E+7* |  | *3.2E+7* |  | *1.9E+9* |  | *1.2E+9* |  | *4E+14* |
| Mass | LUM | MSL | 0.02 | 0.01 | 0.03 | 0.03 | 0.02 | 0.03 | 0.03 | 0.03 | 0.02 | 0.02 | 0.04 | 0.02 | **0.009*** | 0.003 |
|  | LUM | Variance | **0.001*** | 0.01 | **0.0005*** | 0.02 | **0.003*** | 0.02 | **0.001*** | 0.02 | 0.007 | 0.01 | **0.0008*** | 0.02 | **0.002*** | 0.004 |
| PC1 | LUM | MSL | 0.01 | 0.01 | 0.02 | 0.03 | 0.02 | 0.01 | 0.03 | 0.01 | **0.02*** | 0.004 | 0.001 | 0.01 | 0.003 | 0.001 |
|  | LUM | Variance | **0.002*** | 0.01 | 0.01 | 0.02 | 0.01 | 0.009 | 0.02 | 0.009 | 0.002 | 0.005 | 0.01 | 0.009 | 0.007 | 0.001 |
| PC2 | LUM | MSL | 0.002* | 0.0002 | **0.0003^#^** | 0.02 | 0.0004 | 0.002 | **0.0003^#^** | 0.01 | 0.003 | 0.0003 | 0.005 | 0.002 | 0.0009 | 0.002 |
|  | LUM | Variance | 0.008 | 0.01 | 0.008 | 0.004 | 0.01 | 0.02 | 0.009 | 0.009 | 0.007 | 0.01 | 0.01 | 0.02 | 0.002 | 0.0007 |

*: Observed minimum segment-length ratios greater than or observed variances smaller than 95% of the expected values

^#^: Observed minimum segment lengths smaller than 95% of the expected values

boldface: p < 0.05 after Bonferroni sequential adjustments

C is the number of unique simulation ensembles that can be randomly assembled from the source pool (see text for details)
