# Supplementary material for: The Relative Influence of Competition and Prey Defenses on the Phenotypic Structure of Insectivorous Bat Ensembles in Southern Africa
Source: PLoS One. 2008 Nov 13;3(11):e3715. doi: 10.1371/journal.pone.0003715 (PMC2579324; doi:10.1371/journal.pone.0003715)
Supplement: Table S3 — Observed and expected segment-length ratio indices-minimum segment-length (MSL) and variance of segment length ratios-of body size (mass) and principal component (PC1 and PC2) parameters of clutter (CLUT), and open-air (OPEN) bats caught in the fynbos (AL, DHP), forest, (KN), Nama-Karoo (GH, KB), and savanna (SU) ensembles. (0.10 MB DOC) [file pone.0003715.s003.doc]

**Table S3** Observed and expected segment-length ratio indices - minimum segment-length (MSL) and variance of segment length ratios - of body size (mass) and principal component (PC1 and PC2) parameters of clutter (CLUT), and open-air (OPEN) bats caught in the fynbos (AL, DHP), forest, (KN), Nama-Karoo (GH, KB), and savanna (SU) ensembles.

|  |  |  | AL | | DHP | | GH | | KB | | SU | | | |
| --- | --- | --- | --- | --- | --- | --- | --- | --- | --- | --- | --- | --- | --- | --- |
|  |  |  | CLUT | | CLUT | | CLUT | | CLUT | | CLUT | | OPEN | |
| Parameters | Source Pool | Index | Obs | Exp | Obs | Exp | Obs | Exp | Obs | Exp | Obs | Exp | Obs | Exp |
| Mass | Biome | MSL |  |  |  |  | 0.02 | 0.05 | 0.2 | 0.09 | 0.02 | 0.02 |  |  |
|  | Biome | Variance |  |  |  |  | 0.008 | 0.06 | **0.0001*** | 0.06 | **0.007*** | 0.02 |  |  |
| PC1 | Biome | MSL |  |  |  |  | 0.03 | 0.02 | 0.03 | 0.02 | 0.02 | 0.01 |  |  |
|  | Biome | Variance |  |  |  |  | 0.004 | 0.004 | 0.002 | 0.004 | 0.4 | 0.008 |  |  |
| PC2 | Biome | MSL |  |  |  |  | 0.04 | 0.01 | 0.009 | 0.01 | 0.005 | 0.003 |  |  |
|  | Biome | Variance |  |  |  |  | 0.009 | 0.01 | 0.04 | 0.01 | 0.006 | 0.008 |  |  |
| *C* |  |  |  |  |  |  |  | *35* |  | *35* |  | 3003 |  |  |
| Mass | SA | MSL | 0.02 | 0.1 | 0.07 | 0.2 | 0.02 | 0.1 | 0.2 | 0.1 | 0.02 | 0.02 | **0.08*** | 0.02 |
|  | SA | Variance | 0.007 | 0.06 | 0.006 | 0.08 | 0.008 | 0.05 | **0.0001*** | 0.05 | **0.007*** | 0.02 | 0.007 | 0.03 |
| PC1 | SA | MSL | 0.003 | 0.08 | 0.04 | 0.1 | 0.03 | 0.02 | 0.03 | 0.04 | 0.02 | 0.01 | 0.3* | 0.1 |
|  | SA | Variance | 0.003 | 0.02 | 0.03 | 0.04 | 0.004 | 0.01 | 0.002 | 0.02 | 0.4 | 0.008 | 0.8 | 0.04 |
| PC2 | SA | MSL | 0.04 | 0.03 | 0.02 | 0.07 | 0.04 | 0.01 | 0.009 | 0.01 | 0.005 | 0.003 | 0.09 | 0.05 |
|  | SA | Variance | 0.06 | 0.02 | 0.8 | 0.05 | 0.009 | 0.01 | 0.04 | 0.01 | 0.006 | 0.008 | 0.5 | 0.02 |
| *C* |  |  |  | *330* |  | *330* |  | *1820* |  | *1820* |  | *6435* |  | *56* |
| Mass | LUM | MSL | 0.02 | 0.08 | 0.07 | 0.2 | 0.02 | 0.1 | 0.2 | 0.1 | 0.02 | 0.02 | 0.08 | 0.08 |
|  | LUM | Variance | 0.007 | 0.05 | 0.006 | 0.05 | 0.008 | 0.05 | **0.0001*** | 0.05 | **0.007*** | 0.02 | 0.007 | 0.05 |
| PC1 | LUM | MSL | 0.003 | 0.02 | 0.04 | 0.1 | 0.03 | 0.03 | 0.03 | 0.03 | 0.02 | 0.02 | 0.3* | 0.05 |
|  | LUM | Variance | 0.003 | 0.001 | 0.03 | 0.04 | 0.004 | 0.006 | 0.002 | 0.005 | 0.4 | 0.07 | 0.8 | 0.2 |
| PC2 | LUM | MSL | 0.04 | 0.1 | 0.02 | 0.05 | 0.04 | 0.05 | 0.009 | 0.05 | 0.005 | 0.009 | 0.09 | 0.04 |
|  | LUM | Variance | 0.06 | 0.03 | 0.8 | 0.05 | 0.009 | 0.01 | 0.04 | 0.01 | 0.006 | 0.003 | 0.5 | 0.067 |

*: Observed minimum segment-length ratios greater than or observed variances smaller than 95% of the expected values

^#^: Observed minimum segment lengths smaller than 95% of the expected values

boldface: p < 0.05 after Bonferroni sequential adjustments

C is the number of unique simulation ensembles that can be randomly assembled from the source pool (see text for details)
